# Supplementary material for: Levels of and determinants for physical activity and physical inactivity in a group of healthy elderly people in Germany: Baseline results of the MOVING-study
Source: PLoS One. 2020 Aug 13;15(8):e0237495. doi: 10.1371/journal.pone.0237495 (PMC7425978; doi:10.1371/journal.pone.0237495)
Supplement: Attachment — Submitted filename: Comments to the author.doc [file pone.0237495.s002.doc]

Abstract

1. This line is confusing, rewrite using the terms light, moderate and vigorous

*“Insufficient physical activity (PA) and high levels of physical inactivity (PI)”*

Introduction

1. The introduction does not have a clear structure. I suggest the following structure:

| Manuscript   - Effect of insufficient PA - Definition PI or sedentary behavior - Effect of insufficient PA and PI - Effect of regular PA - General prevalencia (disease) - Recomendations (moderate PA) - Global prevalence (recommendations moderate PA) - Germany prevalence (recommendations moderate PA) - Prevalence sedentary - Factors affecting amount of PA and PI - Prevention | Suggestion   - Definition of physical activity (PA) - Effect of regular PA - Recommendations (moderate PA) - General prevalence (recommendations moderate PA) - Germany prevalence (recommendations moderate PA) - General prevalencia (disease) - Definition PI or sedentary behavior - Effect of insufficient PA and PI - Prevalence sedentary - Factors affecting amount of PA and PI - Prevention |
| --- | --- |

2. The use of definitions is not clear, please use light PA, moderate PA and vigorous PA or low or high levels of PA in the introduction section.

3. Explained in detail the background studies in introduction section.

*“Overweight and obesity (BMI>30 Kg/m2 115 ) have an negative influence on the level of PA and PI 116 in the elderly. Higher education has a positive influence on PA. Further factors that influence 117 the amount of PA and PI are marital status, income, wellbeing, psychosocial variables such as 118 self-efficacy, and social and cultural parameters (29,30,35,36)*

*A report from the WHO about the prevention of non-communicable-diseases (NCD) in south120 eastern European countries showed that the promotion of PA and decrease reduction of PI 121 are key aspects in public health efforts (37)”*

Materials and Methods

1. Rewrite the following paragraph including the variables considered in the regression, independent and dependent variables.

*“Descriptive statistics were used to describe the population with respect to the levels of PA and 204 PI. To identify determinants of PA and PI, multiple linear regression models were calculated. 205 All variables were checked for multicollinearity using a correlation (Pearson)”*

Discussion

1-Organize the discussion section based on levels of PA.

Discussion section requires more work; in particular, you must interpret the models you obtained from each regression, then you will be able to compare your interpretations with previous studies.

2. Why is this relevant to the discussion?, this is information should be included in material and methods section.

*“The participants showed a very good adherence in wearing the accelerometer device. The 272 mean wearing time per day was 14 hours. Only 22 of 225 participants had to be excluded from 273 the analysis because of insufficient wearing time”*

3. Did you make any intervention in this study?. Your participants got feedback about their PA, but you do not have a control measure before wear the accelerometer. I think this paragraph should be eliminated.

*“In recent years, the number of interventions targeting PI has increased. However, there is still 294 a lack ofrecommendations for PI (49). In addition, it is also not conclusively established 295 whether PI is an independent risk factor for chronic diseases (49). In summary, recent literature 296 points out that public health activities should emphasise increasing PA at any intensity 297 especially in the elderly. (50)”*

Minor suggestions:

Doble parentheses líne 63

Misplaced reference line 66 (7,8)

Error in parentheses líne 69

Doble parentheses line109

Error in word “therof” líne 221
