# Supplementary material for: Levels of and determinants for physical activity and physical inactivity in a group of healthy elderly people in Germany: Baseline results of the MOVING-study
Source: PLoS One. 2020 Aug 13;15(8):e0237495. doi: 10.1371/journal.pone.0237495 (PMC7425978; doi:10.1371/journal.pone.0237495)
Supplement: Attachment — Submitted filename: Response to Editor.docx [file pone.0237495.s003.docx]

Dear Dr Kleinke,

Although the reviewer who viewed the original manuscript says that the article must be accepted, I sent the revised manuscript to another reviewer, and she found some details. I agree with her that the discussion section should include more interpretive work.

Thank you for your comment. We followed the reviewer comments and added to the discussion section additional information (e.g. the importance of modifiable risk factors for physical and cognitive outcomes as well as more interpretive work related to the results of the regression models). Please see our detailed answer to your comment below.

I also think that phrases such as "Overall, women were slightly more physically active than male participants (mean 1,929.4 min 228 and 1,821.2 min per week, respectively)" include some degree of imprecision because the authors did not use any statistical analysis to make these comparisons; therefore, they found no significant differences (in this case, between genders). I recommend using statistical analyzes of comparison of means (and consider the multiplicity of tests to adjust its level of significance).

Thank you for your comment. We analyzed the mean differences between women and men according to overall PA and found no significant difference. Therefore, we rewrote the section above (please see lines 241-243 in the manuscript).

Perhaps the Introduction, and especially the Discussion, could be enriched if the physical activity were considered as one of the proxies of the Cognitive Reserve (Stern, 2009). Aging is not only related to cardiovascular disease; aging is the principal risk-factor to MCI and dementia. Therefore, I believe that the authors will agree that, in aging, it is not only desirable that the cardiovascular system remains healthy, but also cognitive functions.

Thank you for your comments to the concept of cognitive reserve. So far, our manuscript focused primary on effects of regular PA on physical outcomes (e.g. CVD, mortality or diabetes). Our study was a cooperation with the German Centre for Cardiovascular Research (DZHK). Due to this fact, the primary focus on physical health outcomes can be explained.

Obviously, you are right and PA has also several positive effects on cognitive skills. Therefore, an overall perspective on several physical and physiological dimensions is necessary and important. To address your suggestion, we added the following sentences to the introduction section: “ *(…) and is considered as one of the proxies of the concept of cognitive reserve (8). One of the two most significant modifiable risk factors for dementia is PI. Additionally, PA (in particular aerobic exercise) is positively associated with a less age-related reduction of organic brain matter”* (lines 67-70).

According to address effects of PA on cognition, we added the following sentences to the discussion section (lines 314-320): *“Regular PA is positively associated with several physical health outcomes. Besides that, high levels of PA have also the potential to maintain cognitive health over the long term into higher ages (8,9). In general, high levels of PA can reduce incidence of dementia and cognitive restrictions significantly (48). Norton et al. has shown that changeable risk factors such as PA and PI are responsible for around a third of Alzheimer's disease worldwide. In Europe, USA and UK, physical inactivity was the strongest risk factor (49).”*

We also added the following sentence to the conclusion section (lines 359-361): *“Prevalence of dementia and chronic diseases (e.g. CVD) will probably increase in the next decades. Therefore, the relevance of modifiable risk factors as preventive measures will rise.”*

Also, author should check the abbreviation for "minutes".

Thank you for this comment. We checked the manuscript for abbreviations (“min”). Now, we only use abbreviations (“min”) in tables and notes. In the entire paper, we have adapted the use of "minutes".
